# Hair Analysis for Determination of Isoniazid Concentrations and Acetylator Phenotype during Antituberculous Treatment

**DOI:** 10.1155/2012/327027

**Published:** 2012-10-02

**Authors:** Michael Eisenhut, Detlef Thieme, Dagmar Schmid, Sybille Fieseler, Hans Sachs

**Affiliations:** ^1^Paediatric Department, Luton & Dunstable University Hospital, NHS Foundation Trust, Luton LU40DZ, UK; ^2^Forensisch Toxikologisches Centrum, Munich 80335, Germany; ^3^Institut fur Rechtsmedizin, Ludwig-Maximilians Universität München, Munich 80336, Germany

## Abstract

*Background*. Analysis of isoniazid (INH) uptake has been based on measurement of plasma concentrations providing a short-term and potentially biased view. *Objectives*. To establish hair analysis as a tool to measure long-term uptake of INH and to assess whether acetylator phenotype in hair reflects N-acetyltransferase-2 (NAT2) genotype. *Design and Methods*. INH and acetyl-INH concentrations in hair were determined in patients on INH treatment for *M. tuberculosis* infection using high pressure liquid chromatography/mass spectrometry. Acetyl-INH/INH ratios were correlated with NAT-2 genotype. *Results*. Hair concentrations of INH, determined in 40 patients, were not dependent on ethnic group or body mass index and were significantly higher in male compared to female patients (median (range) 2.37 ng/mg (0.76–4.9) versus 1.11 ng/mg (0.02–7.20) (*P* = 0.02). Acetyl-INH/INH ratios were a median of 15.2% (14.5 to 31.7) in homozygous rapid acetylator NAT-2 genotype and 37.3% (1.73 to 51.2) in the heterozygous rapid acetylator NAT-2 genotype and both significantly higher than in the slow acetylator NAT-2 genotype with 5.8% (0.53 to 14.4) (*P* < 0.05). *Conclusions*. Results of hair analysis for INH showed lower concentrations in females. Acetyl-INH/INH ratios were significantly lower in patients with slow acetylator versus rapid acetylator genotypes.

## 1. Introduction

The first application of hair analysis in monitoring of anti-infective treatment was the measurement of hair concentrations of indinavir, a protease inhibitor, in patients with HIV infection. Indinavir hair concentrations were significantly higher in responders compared to nonresponders [1]. Previous studies analysed antiepileptic drugs in segments of hair of patients on treatment for epilepsy and found that variability of hair drug concentrations, reflecting variable antiepileptic drug ingestion over time, was greater in epileptic patients with sudden unexplained death [2]. Hair analysis in patients on carbamazepine for treatment of epilepsy showed a significant linear relationship between the prescribed dose, hair concentration, and total plasma concentration [3].

Failure of tuberculosis treatment and relapse of tuberculosis, with selection of drug resistance mutations, have not been associated with low maximum concentrations of antituberculosis drugs such as isoniazid (INH). These conditions have been, however, associated with low areas under the concentration curve (AUC) in plasma, which is found in patients with intermittent noncompliance to treatment, widely spaced intermittent therapy, and chronically reduced absorption as found in HIV patients [4–6]. Other factors, which may contribute to subtherapeutic INH concentrations, are age below 5 years and high body mass index [7, 8]. A low AUC for INH has also been associated with the development of acquired rifamycin resistance despite directly observed therapy, leading to isolated rifamycin resistance and multidrug resistant tuberculosis by a lack of protection by INH against resistance development [9]. We introduce the development of hair analysis as a novel tool for quantification of long-term uptake and metabolism of INH. This may help to define the threshold of long-term presence of INH required for effective treatment. Hair analysis has several advantages compared to conventional monitoring of INH drug serum concentrations for generation of an AUC in serum. Hair sampling is painless and not distressing, which is particularly important when children are involved in a study or routine monitoring of INH uptake. INH is not stable in human serum at room temperature unlike in hair. In developing countries with resource limitations, the logistics and expense associated with repeated blood sampling and freezing and transport of blood samples are considerably more challenging compared to obtaining a hair sample with the possibility of storage at room temperature for months if necessary and ease of transport sealed in a plastic bag by ordinary mail to a reference centre where analysis can be performed. The aims of this study were to establish a method for quantification of long-term uptake of INH and the INH acetylator phenotype by measurement of the acetyl-INH/INH ratio in hair samples and to explore determinants of hair concentrations of INH.

## 2. Study Population and Methods

### 2.1. Study Population

Eligible for inclusion into the study were all adults and children commenced on INH treatment for *Mycobacterium tuberculosis* disease or latent infection. Excluded were patients who had received INH treatment prior to the current episode or those who decided to keep their hair very short.

Patients were consecutive patients recruited from adult and paediatric out-patient clinics as well as wards of the Luton & Dunstable University Hospital, NHS Foundation Trust, UK, between 2007 and 2009 and enrolled following informed, written consent by patients and/or parents/legal guardians as appropriate. The majority of patients were recruited from pupils of a school outbreak of *M. tuberculosis* infection described previously [10]. The study had ethical approval by the Hertfordshire Local Research Ethics Committee and approval and full support by the Department of Research and Development of the Luton & Dunstable University Hospital, NHS Foundation Trust. 

Included were patients who agreed not to have their hair cut short for at least 3 months after commencement of treatment.

### 2.2. Hair Analysis for INH and Acetyl-INH

 Hair sampling was done by cutting hair at the occipital hairline. A segment of hair grown during treatment, assuming a growth rate of 1 cm per month [11], was then cut off from the scalp end of the hair sample. 

The hair sample was sent to the Forensic Toxicological Centre in Munich, Germany, and processed there for INH and acetyl-INH concentrations by high pressure liquid chromatography/mass spectrometry: Samples were decontaminated by 5 min agitation in a gas tight tube with 5 mL petroleum benzene (boiling range to 40°C, Merck, Germany), dried and cut into lengths of 1-2 mm. After adding ~50 ng of the internal standard (INH propionate) and 2 mL of formic acid (98–100%, technical grade, AppliChem, Germany), the samples were extracted and derivatised in gas tight tubes by 3 h ultrasonification at 55°C. Respective signals to noise ratios were for INH (as formyl-INH) = 17.2 and Acetyl-INH = 11.8. Similarly, the average signal intensity in 7 blank hair samples (from participants not exposed to isoniazid) corresponded to INH = 0.015 ng/mg and Ac-INH = 0.009 ng/mg.

**Figure 1:**
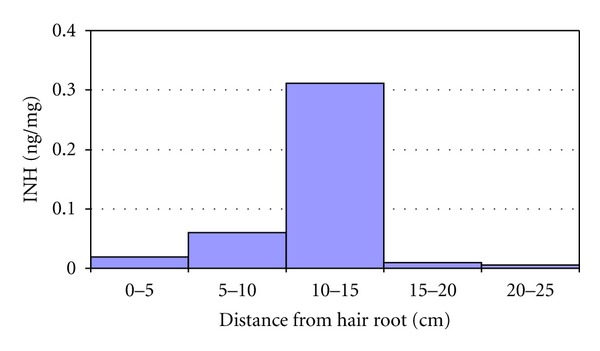
Isoniazid (INH) concentration in the hair shaft as function of distance from the scalp (cm) obtained 9 months after cessation of a 3-month course of treatment.

The linearity of the calibration was confirmed for both analytes within the calibration range, that is, 0.05–1 ng/mg. Relevant correlation coefficients were formyl-INH = 0.975 and acetyl-INH = 0.989. To demonstrate the suitability of hair analysis for retrospective analysis of INH treatment, segments of the hair strand of one patient were analysed 9 months after a three-month course of treatment (see Figure 1). The isoniazid concentration in hair of this participant directly after treatment was 0.27 ng/mg. The maximum concentration in segments of a hair sample of the same patient obtained 9 months after treatment was 0.31 ng/mg. This supported a lack of leaching of isoniazid over time and its stability in hair. The fact that this concentration was even slightly higher may relate to desiccation of the hair shaft increasing drug concentrations in hair over time. This demonstrated the preservation of INH in hair for months after treatment and correlation of its presence with time of treatment.

### 2.3. Determination of NAT-2 Genotype

Mouth swabs to obtain cells from the buccal mucosa were obtained in duplicate for DNA analysis and sent to the Institut fur Rechtsmedizin, Ludwig-Maximilians Universität München, Munich, Germany, where samples were analysed by PCR for the arylamine N-acetyltransferase-2 (NAT-2) genotype.

Genomic DNA was isolated using BioRobot EZ1 system from QIAGEN (Hilden, Germany) [12]. For genotyping NAT2, a fragment of 769 bp was amplified with following primers [13]:

 NAT2f 5′-GAG TTG GGC TTA GAG GCT ATT T-3′
 NAT2fr 5′-TTG GGT GAT ACA TAC ACA AGG G-3′.

The PCR was carried out in a total volume of 25 *μ*L in the presence of 1 ng of genomic DNA as template, 5 pmol of each primer, 10x Gold Buffer (Applied Biosystems, Foster City, CA, USA), 10x dNTP mix, 3 mM MgCl_2_ and 1,25 U *Ampli Taq Gold* (Applied Biosystems, PE Corporation, Foster City, CA, USA). After initial denaturation at 94°C for 12 min, 35 cycles of 30 sec at 94°C, 45 sec at 63°C, and 1 min at 72°C were carried out. The final elongation step was at 72°C for 10 min. 

Afterwards, the PCR products were sequenced by the BigDye Terminator v1.1 Cycle Sequencing Kit (Applied Biosystems, PE Corporation, Foster City, CA, USA) according to the manufacturer's instructions.

### 2.4. Statistical Analysis

Statistical analysis was by Mann-Whitney *U*-test for comparison of continuous data with nonparametric distribution and Pearson and Spearman's rank correlation coefficients were used in calculation of linear and nonlinear correlations as appropriate. Multiple comparisons of nonparametric data were performed by the Kruskal Wallis test and simultaneous multivariate linear regression analysis was conducted to determine which factor independently predicted INH hair concentrations. The software used was SPSS release 18.0. A *P* value of <0.05 was taken to indicate a statistically significant difference.

## 3. Results

### 3.1. Study Population

We recruited a total of 40 patients. 13 were treated for tuberculosis disease and 27 for latent *M. tuberculosis* infection. The mean age was 9.8 years (range: 1.3–29 years). Thirty-one female and nine male patients were recruited. There were 16 Caucasian patients, 14 Asian patients, 9 African or Afro-Caribbean patients, and 1 with unknown ethnic origin. All patients were on an equivalent of 5 mg/kg/day of INH. Treatment for active disease was with INH/rifampicin/pyrazinamide with or without ethambutol in the intensive (2 months) and INH and rifampicin in the continuation phase (4 months). Patients treated for latent *M. tuberculosis* infection were treated with three months of INH and rifampicin. In none of these patients was there any concern about compliance which was directly supervised and documented by the parents in the children (all but two participants) of this study.

### 3.2. Relation of INH Concentrations and Acetyl-INH/INH Ratios to Patient Characteristics

**Figure 2:**
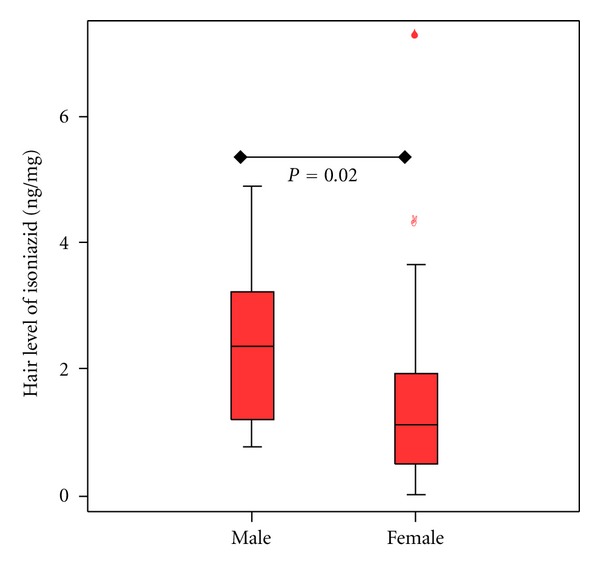
Isoniazid concentration in hair and gender.

**Figure 3:**
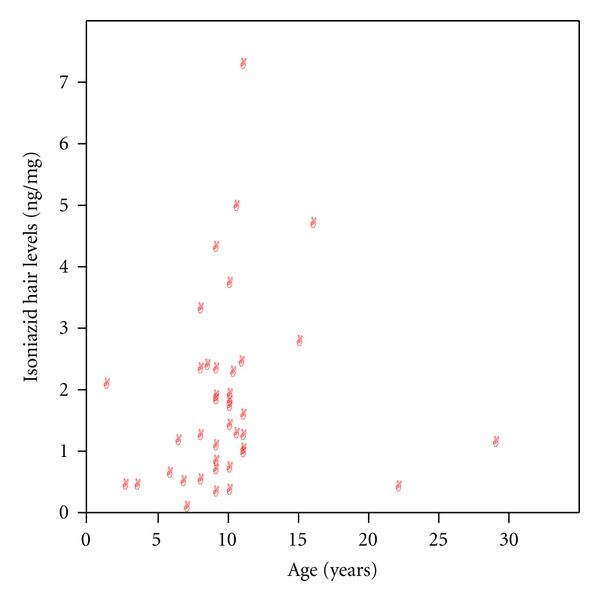
Correlation of isoniazid concentration in hair and age.

**Figure 4:**
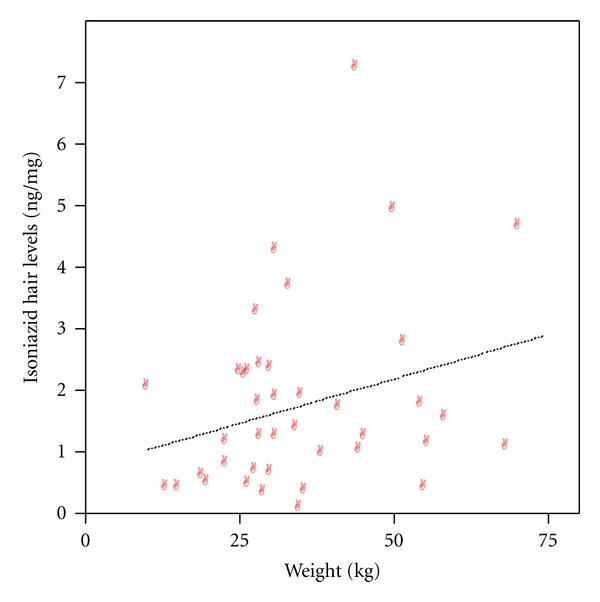
Correlation of isoniazid concentrations and body weight (regression line inserted).

Investigation of the relationship of gender and INH concentrations and acetyl-INH/INH ratios revealed that INH concentrations were with a median of 2.37 ng/mg (range 0.76–4.9) significantly higher in male compared to female patients with 1.11 ng/mg (0.02–7.20) (*P* = 0.02) (see Figure 2). Acetyl-INH/INH ratios were not different between genders with a median of 11.5 (range 0.74–51.22) in male and 6.7 (0.48–47.5) in female patients (*P* = 0.32). Correlation of age with INH concentrations revealed a significant correlation with *r* = 0.28 (Spearman's rank correlation coefficient) (one-tailed *P* = 0.04) (see Figure 3) and weight with *r* = 0.275 (Pearson's correlation coefficient) (one-tailed *P* = 0.043) (see Figure 4). 

Comparison of the predictive power of age and gender in determination of INH concentrations by simultaneous multiple linear regression analysis revealed that gender was with *t* = −1.79 (*P* = 0.081) a better predictor than age with a *t* = 0.55 (*P* = 0.58) but none of these patient characteristics was significantly associated in this analysis. There was no correlation of INH concentrations with BMI (*r* = 0.213, *P* = 0.1). There was also no correlation of acetyl-INH/INH ratio and weight or age, (*P* = 0.356 and 0.276, resp., by Spearman's rank correlation coefficient). Comparison of INH concentrations between African, Asian, and Caucasian ethnic groups revealed a median of 1.66 ng/mg (range 0.02–7.20) in African patients, 0.83 (0.27–2.72) in Asian, and 1.74 (0.3–4.23) in Caucasian patients and there was no significant difference between groups (*P* = 0.130). Comparison of INH hair concentrations in patients with active versus latent *M. tuberculosis* infection revealed that there was with a median of 1.76 ng/mg (range 0.35–4.6) in patients with active tuberculosis no significant difference from hair concentrations of patients with latent *M. tuberculosis* infection of 1.19 (0.02–7.2) (*P* = 0.66).

### 3.3. Relationship of Acetylator Phenotype and Genotype

**Figure 5:**
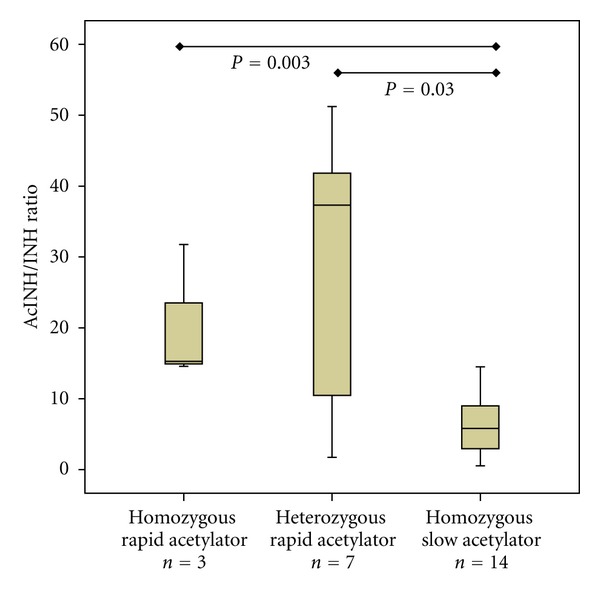
Acetylator phenotype represented as acetylisoniazid/isoniazid ratio (AcINH/INH ratio) and arylamine N-acetyltransferase 2 genotype (*n* = 24).

Acetylator phenotype represented as acetyl-INH/INH ratio was with a median of 15.2 ng/mg (range 14.5–31.7) in patients with a homozygous rapid acetylator genotype and with a median of 37.3 (1.73–51.2) in patients with heterozygous rapid acetylator genotype significantly higher than in patients with a slow acetylator genotype of 5.8 (0.53–14.49) (*P* = 0.013) (see Figure 5). The difference between homozygous rapid acetylator and heterozygous rapid acetylator genotype was not statistically significant (*P* = 0.517). There was no significant correlation between INH concentrations in all patients and acetyl-INH/INH ratio (*r* = 0.1, *P* = 0.538). INH concentrations in patients with homozygous rapid acetylator genotype had a median of 0.36 ng/mg (range 0.02–7.2), in patients with heterozygous rapid acetylator genotype 0.90 ng/mg (0.56–4.9) and in patients with slow acetylator genotype 1.91 ng/mg (0.27–3.23) and the difference between the groups was not significant (*P* = 0.503). There was no difference in age, gender, or weight in different acetylator genotypes.

## 4. Discussion

To our knowledge, we present the first data on hair concentrations of INH and its determinants. We demonstrated that hair analysis can be used to analyse INH uptake and acetylator phenotype. Results of this acetylator phenotype analysis were validated by analysis of the acetylator genotype. The wide variability of acetylator phenotypes within the heterozygous rapid acetylator genotype has been found previously on analysis of the acetyl-INH/INH ratios in peripheral blood [14]. We did not find a significant correlation of acetylator phenotype or genotype and INH hair concentrations. This may be due to the fact that group sizes were small. INH concentrations increased in ascending order from homozygous rapid over heterozygous rapid to slow acetylator genotype, but this was not significant. A previous study investigating areas under the concentration—time curve for INH during 12 hours after dosing—found a highly significant association with NAT-2 genotype [6]. The lack of association of age with acetylator phenotype in hair supported results of a previous study showing that the acetylator phenotype calculated from the acetyl-INH/INH ratio in plasma 3 hours after dosing is age dependent, that is increases with age up to the age of 4 years, but is not age-dependent after this age [15]. In our study, only 3 patients were younger than 5 years of age. We found that on univariate analysis gender was significantly associated with hair INH concentrations. A previous investigation demonstrated that the half-life of INH in serum was significantly longer in males compared to female patients [16]. This may provide a mechanism for increased accumulation of INH in hair of male patients over time found in our study. In a study conducted in India, more patients with low concentrations four and a half hours after administration were found in female compared to male patients independent of age and body weight [17]. In contrast to these results, a more recent investigation showed a significantly higher area under the INH concentration curve for female adults compared to male adults in the 8 hours after application of INH [18]. Another recent investigation showed no significant gender difference in INH concentrations in plasma one or four hours after INH application or in epithelial lining fluid or alveolar cells four hours after application [19].

With regards to age dependency of INH concentrations a previous cohort study in South African children found that each year increase in age was associated with increases in INH peak concentrations of 6% [20] but age was noncontributory to a multivariate analysis model describing the effects of genotype and dose in mg/m^2^. AUCs for INH in children in another study showed, on within genotype regression, a significant increase with age [7]. In our study, the contribution of age was not statistically significant in a multivariate model. 

The significantly faster elimination of INH by infants and younger children has been ascribed to the relatively greater mass of the liver in proportion to total body weight and it has been proposed that more optimal doses would be calculated on the basis of body surface area rather than body weight.

Body weight was not associated with INH hair concentrations on multivariate analysis. A previous investigation noted a positive correlation of body weight with plasma INH concentrations [21].

The fact that INH hair concentrations were not different in cases of active tuberculosis compared to latent *M. tuberculosis* infection indicates that active tuberculosis was not associated with malabsorption of INH, a phenomenon postulated for patients with active tuberculosis previously [22]. 

Previous studies noted an enhanced drug uptake of darker hair due to its higher melanin content [23]. Our study found no difference between ethnic groups making it unlikely that hair colour is a significant factor in hair concentrations of INH. 

Hair analysis is uniquely placed to investigate the causes of treatment failure or relapse in and outside a trial setting. Hair analysis can offer identification of causes of disease relapse to establish whether it is due to a loss of pharmacological efficacy (drug resistant strain) or noncompliance. It also overcomes the limitations of plasma monitoring in identifying patients who, following periods of total noncompliance, comply with the prescribed dose a few days prior to clinical visits so as to achieve respectable steady state concentrations [3].

A low INH concentration in the hair of a patient with treatment failure could be interpreted as follows.

- By the analysis of segments of a hair shaft increased variability of INH concentrations between segments indicates poor compliance.
- A decreased INH/acetyl-INH ratio indicates homo- or heterozygous rapid acetylator status.
- A uniformly low INH concentration in all segments of a hair shaft in the absence of a low INH/acetylINH ratio, age, or weight could indicate malabsorption.

## 5. Conclusions

Results of hair analysis for INH showed lower concentrations in females. Acetyl-INH/INH ratios were significantly lower in patients with slow acetylator versus rapid acetylator genotypes.
